# Corrigendum

**DOI:** 10.1111/jcmm.13094

**Published:** 2017-01-25

In Nyman *et al*. 1, an error occurred in Figure 1 Panel B. The G3PDH immunoblot presented for HEK293 cells is a duplication of the G3PDH immunoblot related to H82 cells. Correct version of Figure 1 (Panel B) should have been as depicted below:

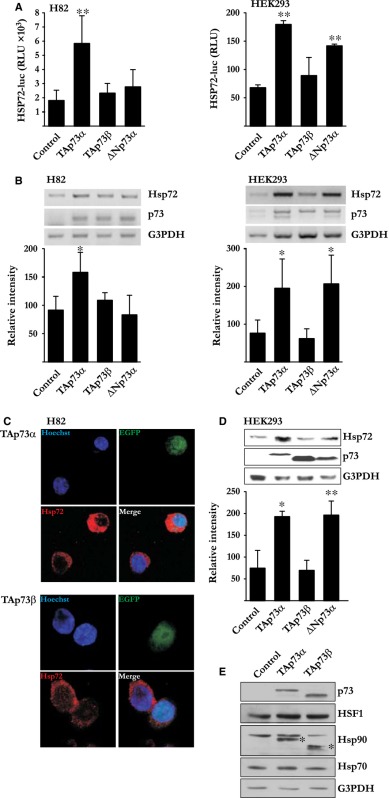

The authors wish to apologize for any misunderstanding or inconvenience caused.
